# Supplementary material for: A Comprehensive Investigation of Stimulatory Agents on MAIT and Vα7.2+/CD161− T Cell Response and Effects of Immunomodulatory Drugs
Source: Int J Mol Sci. 2024 May 28;25(11):5895. doi: 10.3390/ijms25115895 (PMC11172258; doi:10.3390/ijms25115895)
Supplement: Supplementary file 1 [file ijms-25-05895-s001.zip › ijms-3013640-supplementary.pptx]

## Slide 1
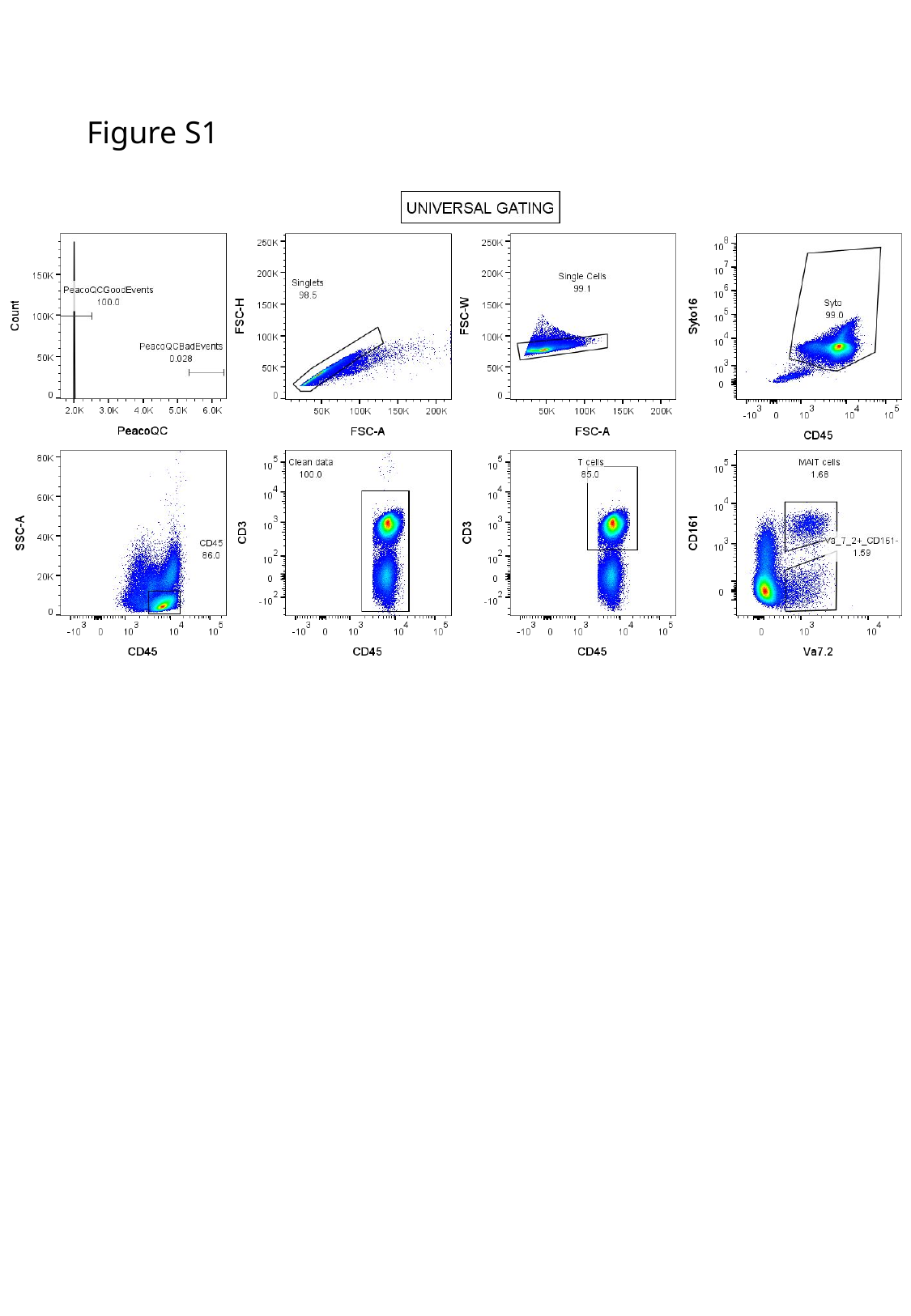

Figure S1

## Slide 2
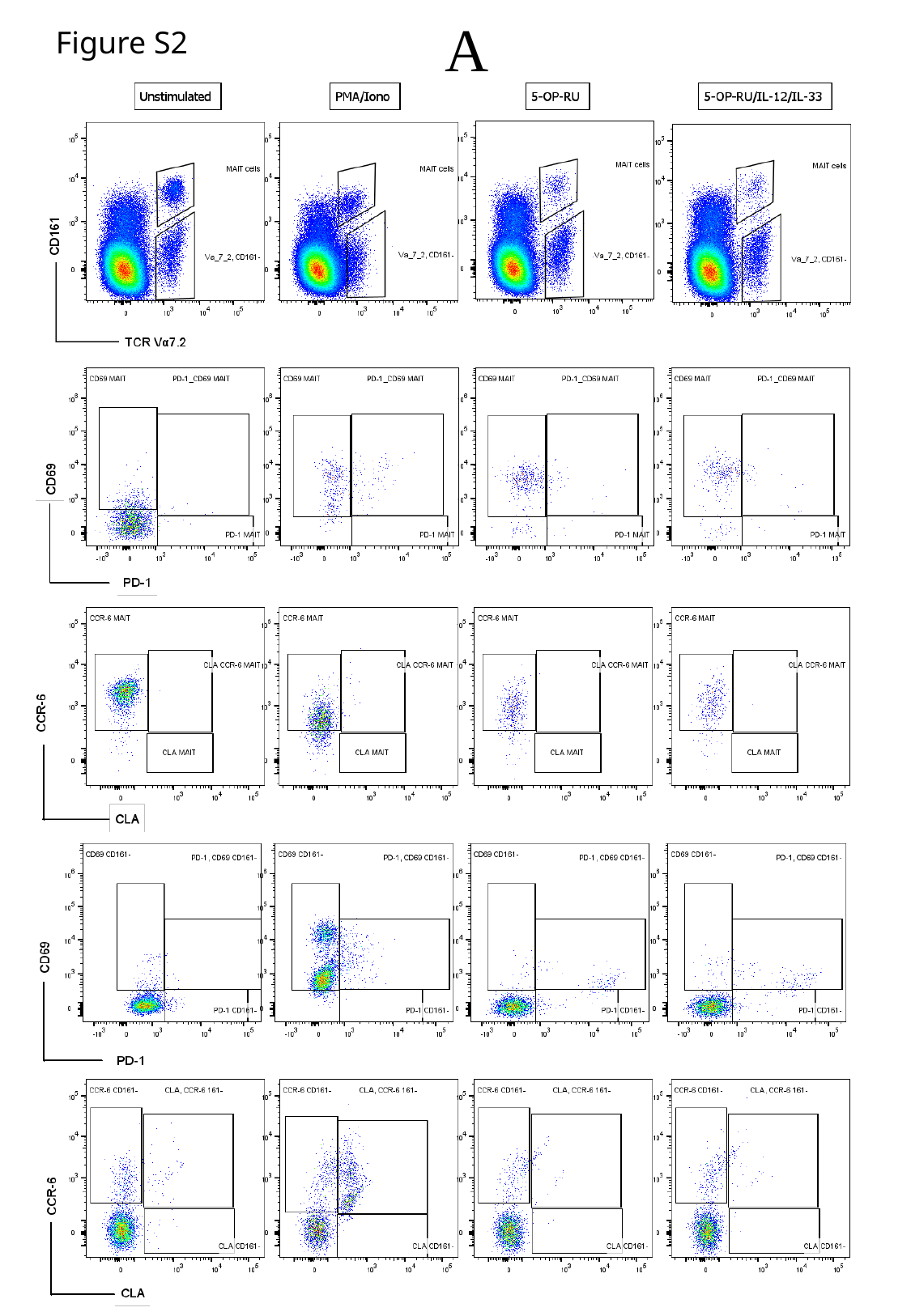

A
Figure S2

## Slide 3
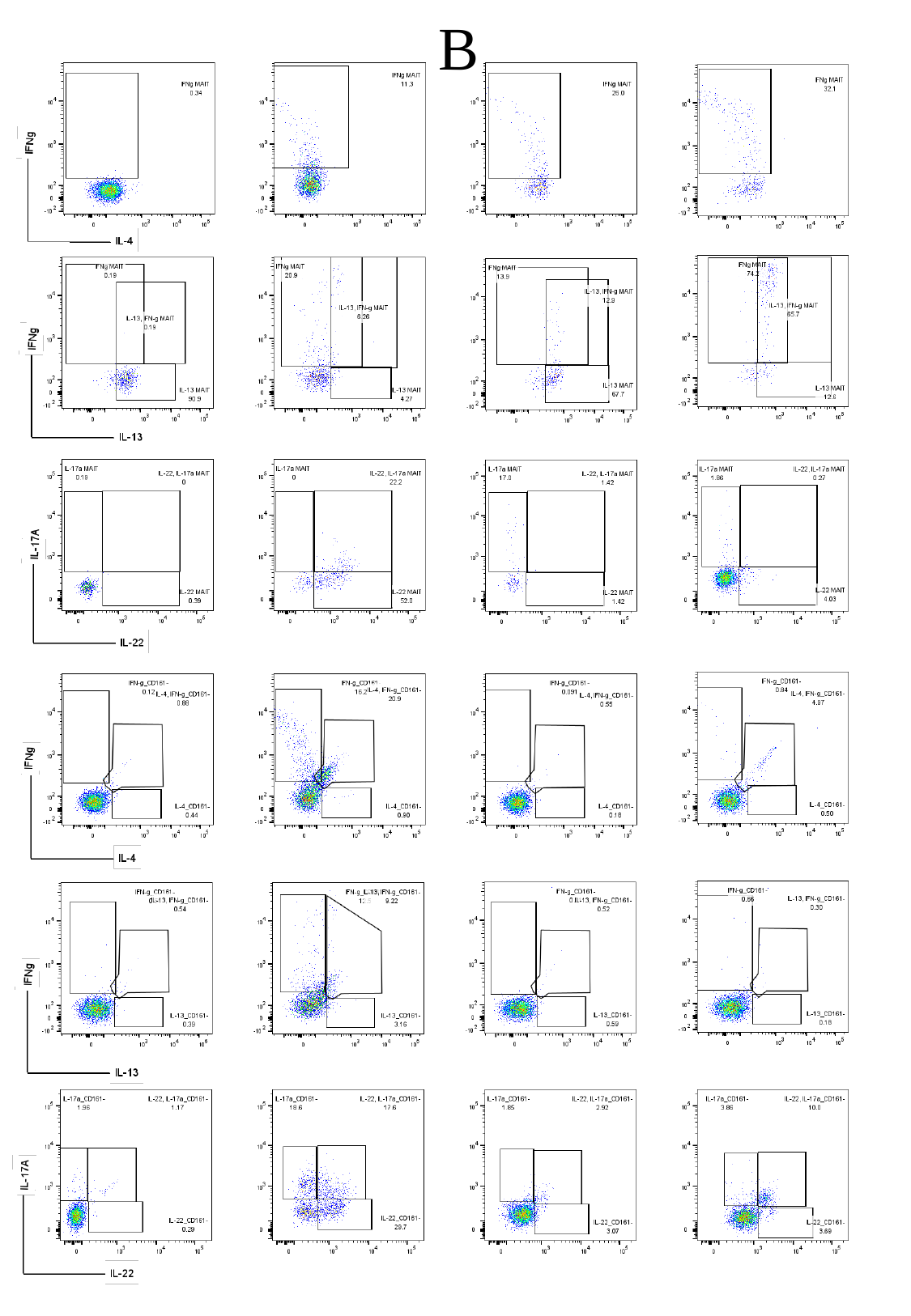

B

## Slide 4
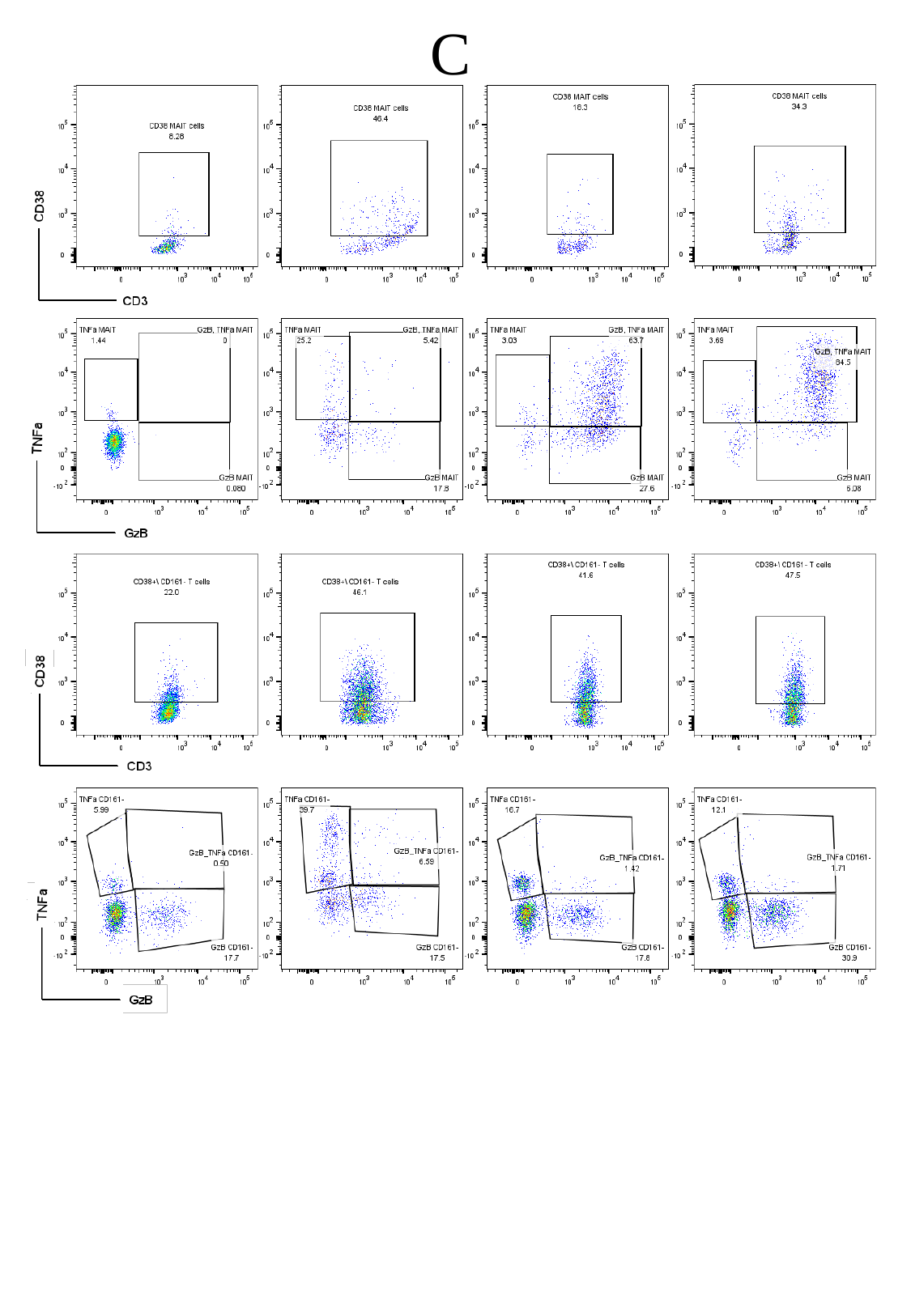

C

## Slide 5
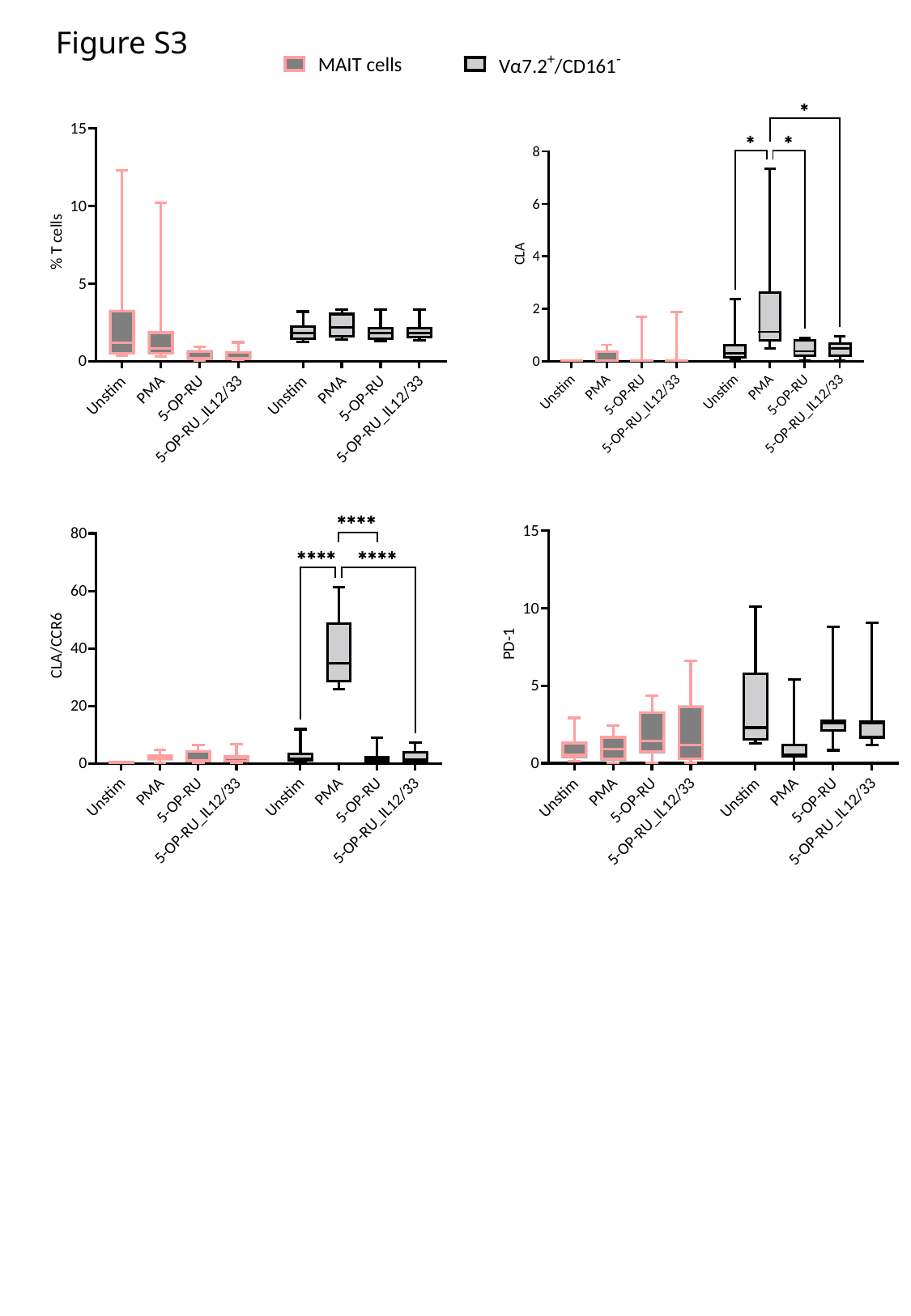

Figure S3

## Slide 6
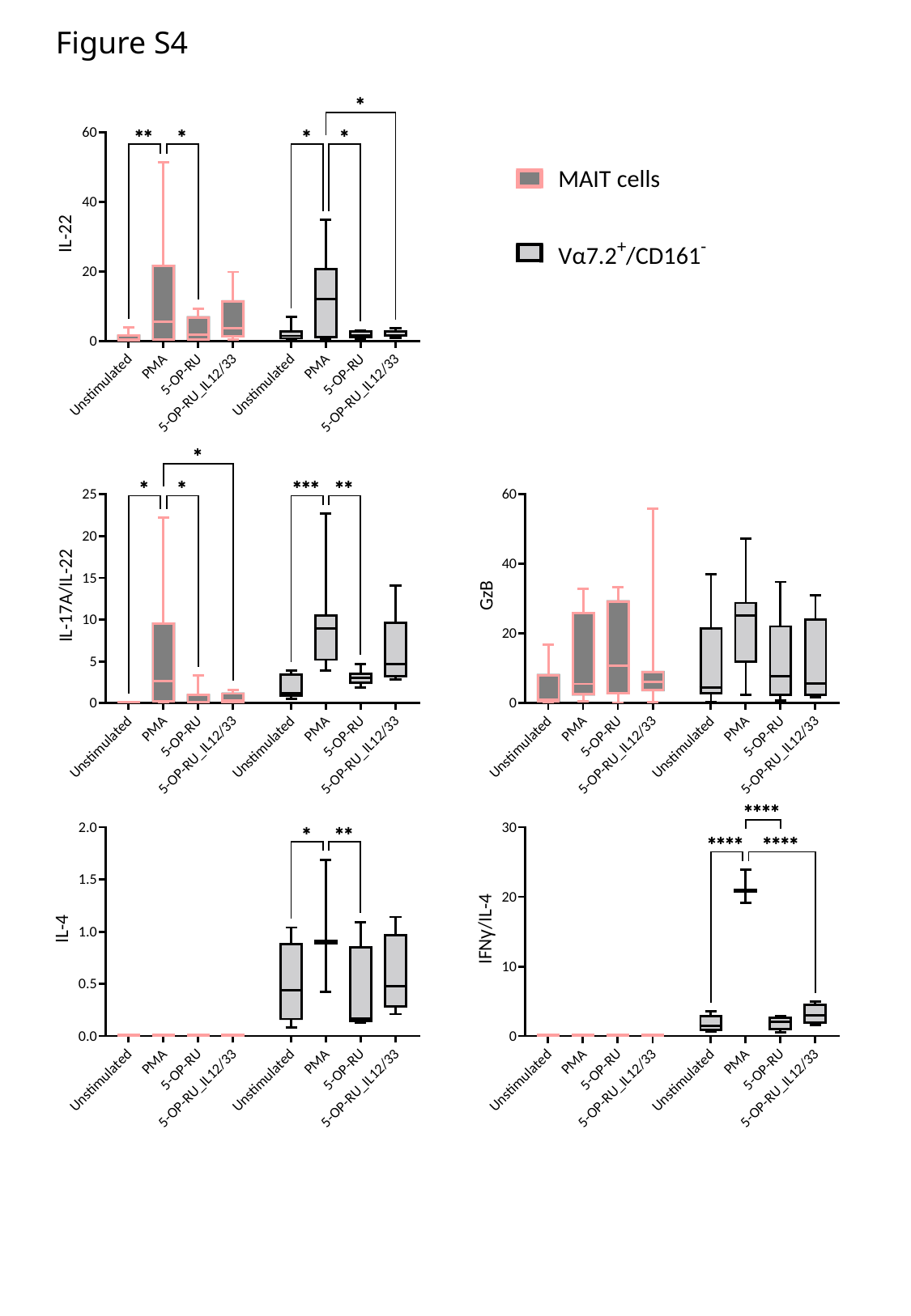

Figure S4

## Slide 7
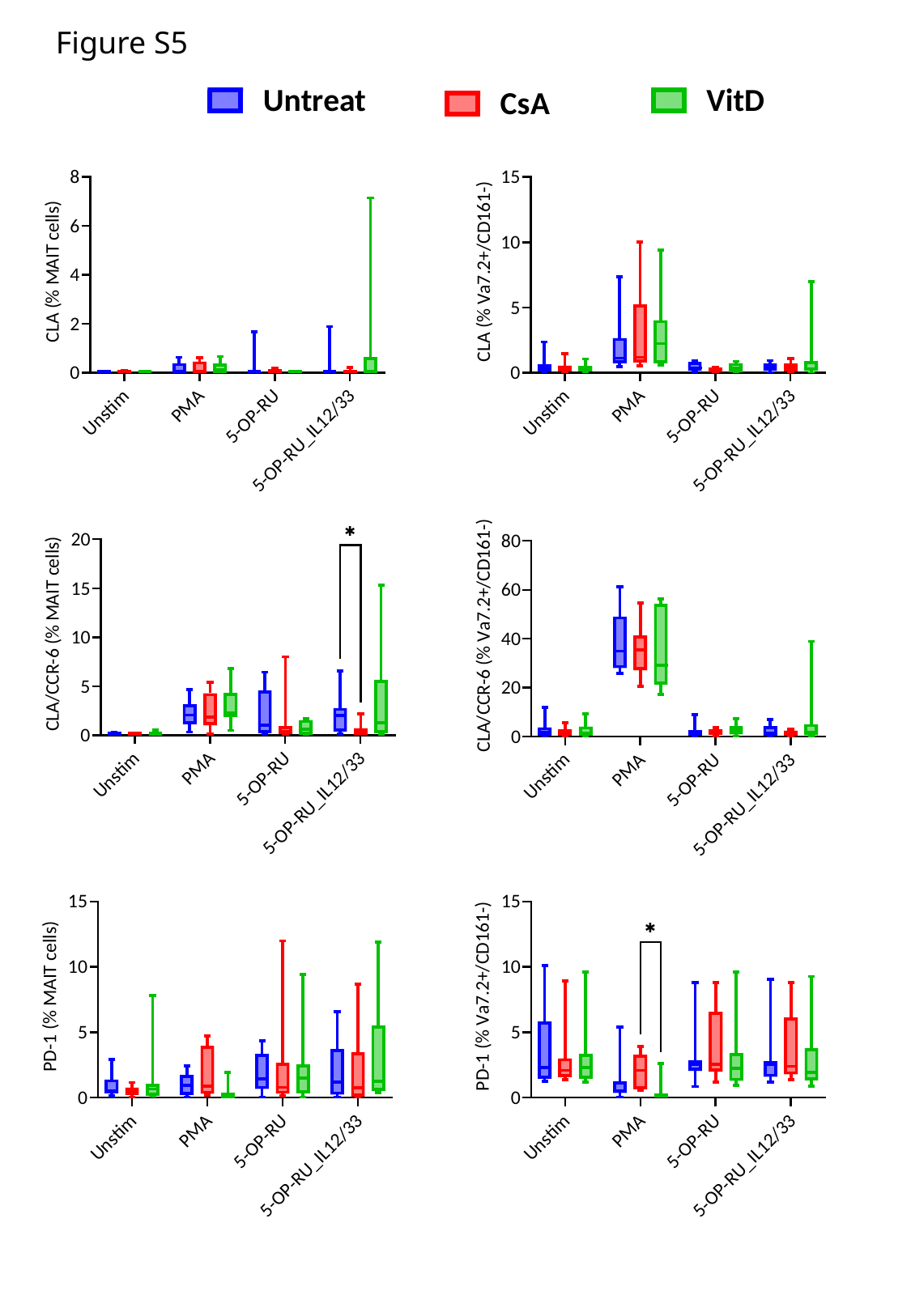

Figure S5

## Slide 8
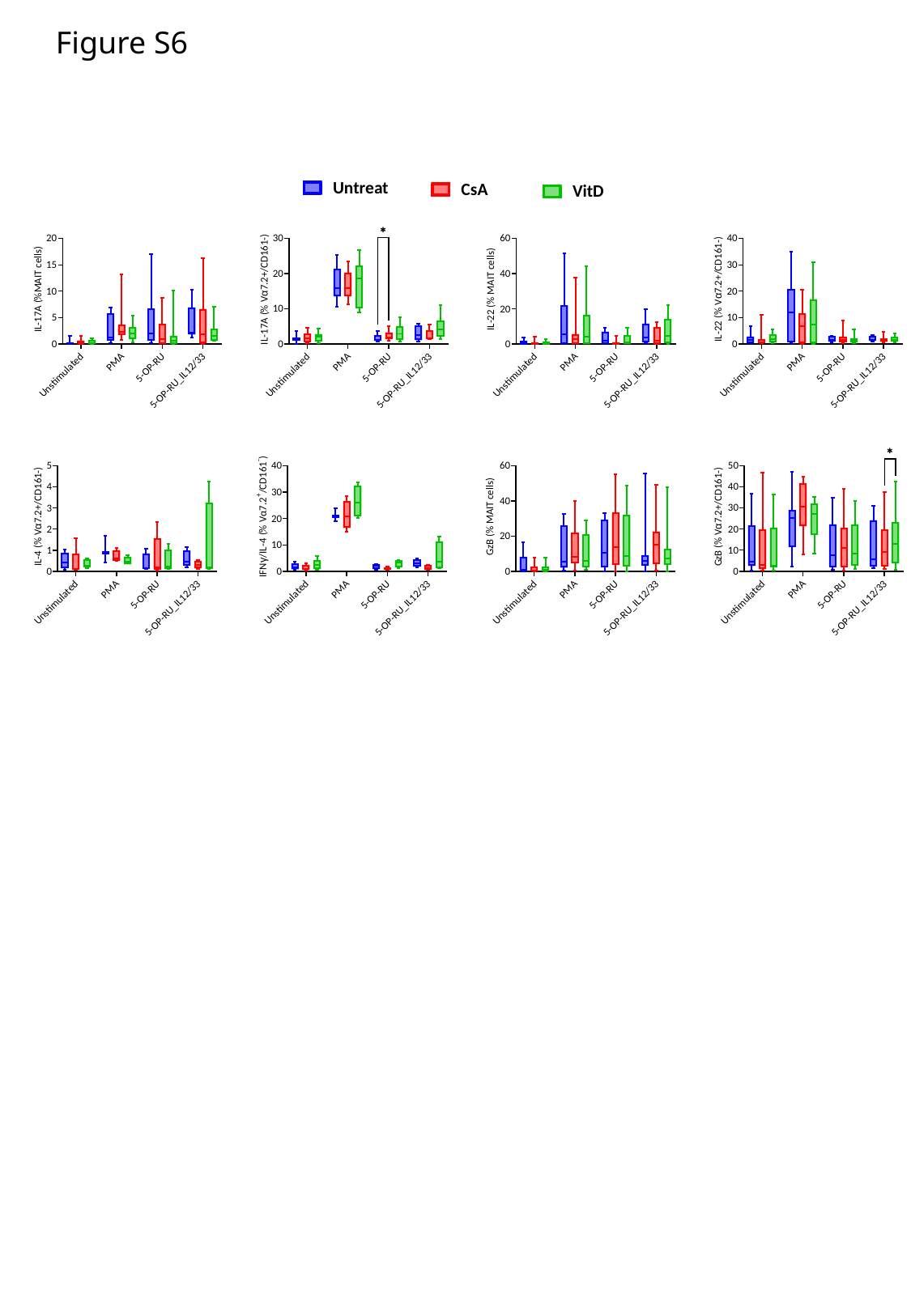

Figure S6
